# Players in Mitochondrial Dynamics and Female Reproduction

**DOI:** 10.3389/fmolb.2021.717328

**Published:** 2021-10-11

**Authors:** Weiwei Zou, Dongmei Ji, Zhiguo Zhang, Li Yang, Yunxia Cao

**Affiliations:** ^1^ Reproductive Medicine Center, Department of Obstetrics and Gynecology, The First Affiliated Hospital of Anhui Medical University, Hefei, China; ^2^ NHC Key Laboratory of Study on Abnormal Gametes and Reproductive Tract (Anhui Medical University), Hefei, China; ^3^ Key Laboratory of Population Health Across Life Cycle (Anhui Medical University), Ministry of Education of the People’s Republic of China, Hefei, China; ^4^ Anhui Province Key Laboratory of Reproductive Health and Genetics, Hefei, China; ^5^ Biopreservation and Artificial Organs, Anhui Provincial Engineering Research Center, Anhui Medical University, Hefei, China; ^6^ Department of Pediatrics, Xiangya Hospital, Central South University, Changsha, China

**Keywords:** mitochondrial dynamics, protein, diseases, therapy, reproduction

## Abstract

Mitochondrial dynamics (fission and fusion) are essential physiological processes for mitochondrial metabolic function, mitochondrial redistribution, and mitochondrial quality control. Various proteins are involved in regulating mitochondrial dynamics. Aberrant expression of these proteins interferes with mitochondrial dynamics and induces a range of diseases. Multiple therapeutic approaches have been developed to treat the related diseases in recent years, but their curative effects are limited. Meanwhile, the role of mitochondrial dynamics in female reproductive function has attracted progressively more attention, including oocyte development and maturation, fertilization, and embryonic development. Here, we reviewed the significance of mitochondrial dynamics, proteins involved in mitochondrial dynamics, and disorders resulting from primary mitochondrial dynamic dysfunction. We summarized the latest therapeutic approaches of hereditary mitochondrial fusion–fission abnormalities and reviewed the recent advances in female reproductive mitochondrial dynamics.

## Introduction

Mitochondria, cellular organelles with bi-membrane structures, are essential for maintaining cell metabolism and producing energy. Mitochondrial dynamics is a continuous process of fission and fusion between the inner mitochondrial membranes (IMMs) and the outer mitochondrial membranes with other mitochondria (Wai and Langer, 2016). Mitochondria effectively modulate fission and fusion to exchange matrix and membrane components (Frederick and Shaw, 2007). The constant and homeostatic fission and fusion processes form a dynamic interconnection network that maintains the integrity of mitochondria and mtDNA (Suen et al., 2008). Impairments in mitochondrial dynamics are often related to cellular energic deficiency, especially in those tissues in high demand of energy, like neurons, cardiomyocytes, and muscle cells. In general, impaired oxidative phosphorylation (OXPHOS), mtDNA deficiency, and overproduction of reactive oxygen species (ROS) under pathological conditions induce mitochondrial fusion (Jendrach et al., 2008; Tondera et al., 2009).

Mitochondrial dysfunction tends to increase ROS production, which can lead to mtDNA mutations and cell damage. Mitochondrial fusion can either improve energy supply through expanded IMMs or promote the material exchange and complementation between healthy and defective mitochondria, ensuring mtDNA integrity and recovery of mitochondrial respiratory function (Liesa and Shirihai, 2013; Cai and Tammineni, 2016). The inhibition of mitochondrial fusion leads to mtDNA loss and declined mitochondrial respiration function (Clark-Walker and Miklos, 1975; Harvey, 2019). Mitochondrial fission leads to the removal of aberrant mitochondria while under stress and undergoing apoptosis and the assignment of mitochondria into daughter cells (Twig et al., 2008) (Chan, 2012). Inhibition of mitochondrial fission prevents repairing of disabled mitochondrial function (Benard et al., 2007; Twig et al., 2008).

Mitochondria are the most abundant organelles in oocytes and embryos. They undergo constant dynamic changes during oocyte maturation and embryonic development before implantation to support major cellular development events (Motta et al., 2000; Sathananthan and Trounson, 2000; Harvey, 2019). Human oocytes metabolize pyruvate *via* OXPHOS, primarily during the growth to provide energy (Harvey, 2019). Human oocytes and early embryo metabolism are characterized by low oxidative metabolism and oxygen consumption and the utilization of pyruvate, lactic acid, and amino acids to support development (Dumesic et al., 2015). The blastocyst stage showed high levels of glycolysis and oxygen consumption. It resulted in the activation of the embryonic genome and higher energy requirements for blastocyst formation (Gardner and Harvey, 2015). Fluctuating energy requirements promote constant variations in mitochondrial dynamics, which dynamically and harmoniously modulate metabolism to support the development of oocytes and embryos. The typical mitochondrial fission–fusion mechanism forms a dynamically interconnected network to preserve regular mitochondria activity. Meanwhile, it satisfies the material and energy demands to maintain oocyte maturation and preimplantation embryonic development (Frederick and Shaw, 2007).

This review aims to summarize the findings of current investigations into mitochondrial dynamics–related proteins, related diseases, targeted therapy, and vital functions of mitochondrial dynamics in oocyte and embryonic development. We found that the research on mitochondrial dynamics in female reproduction is quite limited. Therefore, further research and therapeutic methods will be discovered in the field, which may draw greater attention to mitochondrial dynamics and female reproduction.

## Significant Players of Mitochondrial Dynamics

### Dynamin-Related Protein 1

The most knowable one of these series is DRP1, a mitochondrial fission GTPase (Otera and Mihara, 2011). Also known as DNM1L (Dynamin 1–like), DRP1 is a member of the dynamin family of large GTPases. DRP1 shares a similar architecture with the other dynamin superfamily members. The polypeptide chain folds backward to form a monomer with four domains (the head, neck, trunk, and foot). The head consists of a GTP-binding G domain, the trunk contains a self-organizing interface, and the neck is an essential component of the bundle signal (Reubold et al., 2015; Kalia et al., 2018). The foot is a variable domain containing approximately 100 amino acid residues that bind negative-charged lipids such as cardiolipin and phosphatidic acid (Stepanyants et al., 2015; Adachi et al., 2016). It controls the final part of mitochondrial fission, pinching off the membrane stalk between two daughter mitochondria. Without normal functioned DRP1, the tubular projections of mitochondria typically present in cells are retracted into large perinuclear aggregates (Smirnova et al., 1998). Therefore, pioneering researchers have suggested that DRP1 maintains mitochondrial morphology by distributing the yeast Dnm1 corresponding mitochondrial tubules throughout the cytoplasm. Additional studies have shown that DRP1 plays a role in programmed cell death during *C. elegans* development. DRP1 is also involved in the restructuring and opening of mitochondrial cristae during apoptosis (Wang et al., 2012). Furthermore, DRP1 induces programmed cell death *via* the ligation to CD47, which requires its translocation from cytosol to mitochondria (Kamerkar et al., 2018). Moreover, DRP1 is also essential for maintaining mitochondrial health. In heteroplasmic m.3243 A > G cells, silencing of DRP1 was associated with increased levels of mutant mitochondrial DNA (Malena et al., 2009). Finally, DRP1 has to be precisely modified to functions normally. IFN- β, a mitochondrial fission initiator, phosphorylates STAT5 and upregulates PGAM5, phosphorylating serine 622 of DRP1 (Tresse et al., 2021). Phosphorylated PGAM5S, a complex required during necrosis, recruits DRP1. It activates DRP1 by dephosphorylation and induces mitochondrial fragmentation and execution of necrosis (Wang et al., 2012). Conversely, DRP1 knockdown leads to elongated peroxisomes, indicating a ubiquitous divisome function of DRP1 (Kamerkar et al., 2018). DRP1 needs adaptors to anchor the mitochondrial outer membrane inside the cell. These adaptors were first identified in yeast, including Fis1, Mdv1, and Caf4. There is no homolog in metazoans of Mdv1 and Caf4, and the mammalian has their specific adaptor for DRP1. This series of proteins comprise the mitochondrial fission factor (MFF) (Gandre-Babbe and van der Bliek, 2008) and the chordate-specific mitochondrial dynamics proteins of 49 kDa (MiD49), also known as MIEF2, and 51 kDa (MiD51), also known as MIEF1 (Otera et al., 2016). Those adaptors can recruit DRP1 solely; ablation of their function will lead to dysregulation of mitochondrial fission.

### Inverted Formin 2

INF2, also known as ER-localized inverted formin 2, is a member of the formin family, binds to the barbed ends of developing filaments, and protects them from capping. They create long actin filaments to cross-link into bundles. IFN2 interacts with the formin homology-1 and 2 (FH1 and 2) domain, gelsolin, and profilin to block the capping of the barbed end and create short filaments of actin subunits through a combination of barbed end elongation, severing, and WH2 motif-mediated depolymerization (Bindschadler and McGrath, 2004; Chhabra and Higgs, 2006; Gurel et al., 2015). INF2 was an upstream factor of DRP1. Actin filaments accumulate between mitochondria and INF2-enriched ER membranes at constriction sites. Some research studies indicate that INF2 enrichment might be the initial step of mitochondrial constriction before the accumulation of DRP1 (Korobova et al., 2013). Subsequently, IFN stabilizes the ER–mitochondrial platforms and tethers the damaged mitochondria to the ER, separating them through fission (Tresse et al., 2021). ER–mitochondria contacts increase calcium uptake by mitochondria and mitochondrial division (Steffen and Koehler, 2018). Furthermore, an actin-nucleating protein, Spire 1C, directly links mitochondria to the actin cytoskeleton and the ER. Spire 1C cooperates with IFN2 and promotes actin assembly on the mitochondrial surface, driving one of the initial steps of mitochondrial fission (Manor et al., 2015).

### Mitochondrial Fission 1

FIS1 was first discovered in yeast as a gene capable of relieving temperature-sensitive alleles of the fusion genes (James et al., 2003). Its human orthologue, also known as hFis1, is a component of a mitochondrial complex that promotes mitochondrial fission (James et al., 2003). FIS1, localized to the outer mitochondrial membrane, was first thought to be a helper factor of DRP1 during mitochondrial fission and cellular apoptosis. The C-terminus anchor of FIS1 was crucial for the localization of mitochondria. A 15-kDa soluble domain inside this domain with two tetratricopeptide repeats (TPRs) acts as the tethering site of the mitochondrial outer membrane. The subsequent study found that FS1 recruits DRP1 from the cytosol to the fission site of mitochondria (Ihenacho et al., 2021). Coimmunoprecipitation studies suggest that FIS1 may act as a downstream factor of Mff of DRP1 recruitment and assembly at scission sites (Shen et al., 2014). Knockout of *Fis1* and *Mff* simultaneously resulted in a more pronounced mitochondrial elongation phenotype than knockout of them independently, suggesting an independent role of the above two during mitochondrial fission (Shen et al., 2014).

The number of FS1 molecules is the limiting factor of the frequency of mitochondria fission (Yoon et al., 2003). Although FIS1 plays a significant role in mitochondria fission, overexpression of FIS1 makes no alternation on the membrane potential, PH value, or the calcium cation capacity of mitochondria (Frieden et al., 2004). Like DRP1, attenuation of FIS1 led to the increase in heteroplasmy of pathological m.3243 A > G mutation (Malena et al., 2009). In addition, FIS1, which is also colocalized with DRP1 during peroxisome fission (Kobayashi et al., 2007), may have a role in cellular apoptosis. However, studies in lower animals found controversial results compared to those in mammalian cells. The ablation of Fis1 in *C. elegans* did not elongate mitochondria as expected (Breckenridge et al., 2008), while the embryonic fibroblast of the *Fis1* ^−/−^ mouse presented with elongated mitochondria. Additionally, the knockout of *Fis1* in mice is embryonic lethal (Losón et al., 2013). Many studies have explored the relationship between FIS1 and other proteins that play a role in mitochondrial dynamics. A report proposed that FIS1 may act as the negative regulator of Mid51 while recruiting DRP1 (Zhao et al., 2011). Studies showed that FIS1 was a stress-specific DRP1 recruiter, while Mff was the primary recruiter of DRP1 in mammalian cells (Otera et al., 2010; Otera et al., 2016). On the other hand, Fis1 activated Caspase-8 during cell damage (Iwasawa et al., 2011); furthermore, Fis1 induces hierarchical macroautophagy and inhibition of syntaxin 17, indicating that Fis1 is involved in both mitophagy and apoptosis (Xian et al., 2019). A recent study claimed that Fis1 dominated the peripheral mitochondrial fission, enabling damaged material to be shed into smaller mitochondria, ultimately directing to mitophagy (Kleele et al., 2021). Those pieces of evidence emphasize the stress-reactor role of Fis1. However, the primary function of FIS1 in the cell remains fully elucidated.

### Mitochondrial Fission Factor

MFF is a 342 amino acid protein containing two short repeat motifs close to the N-terminus, followed by a helical coil domain, a transmembrane domain, and a short C-terminal tail. Alternative splicing of MFF results in 19 transcript variants locating either on the cell membrane or the cytosol. Mff was first anchored *via* its tail to the mitochondrial outer membrane with Fis1 in drosophila cells (Gandre-Babbe and van der Bliek, 2008). Activation of adenosine monophosphate (AMP)-activated protein kinase (AMPK) resulted in rapid mitochondrial fragmentation. The substrate screening of AMPK has revealed that Mff catalyzes the guanosine triphosphatase category, which speeds up mitochondrial fission (Toyama et al., 2016). A recent study in mouse cardiomyocytes found that Mff only regulates cellular midzone fission. The fission that occurs in the midzone is unlikely to colocalize with the lysosome. They also found that fission in the peripheral area is frequently combined with lysosome recruiting, suggesting that it is responsible for mitochondria proliferation (Kleele et al., 2021). This research might be the potential explanation for why MFF and FIS1 act so distinctly.

### Mitochondrial Dynamics Proteins of 49 kDa and 51 kDa

Also known as MIEF1 and MIEF2, both have nucleotidyltransferase folds with an N-terminal transmembrane anchor for integration into the mitochondrial outer membrane (Osellame et al., 2016). They share many architectural similarities. However, only MID51 can bind nucleotide diphosphates (ADP and GDP), whereas the ligand binding MID49 is still unknown. MID51 is dimeric; in contrast, MID49 is monomeric, but both share motifs interacting with DRP1 (Losón et al., 2014). Their structural differences might suggest a differential regulation and stabilization of MID51 versus MID49 (Richter et al., 2014; Losón et al., 2015). Different reports have ascribed opposing roles to these two proteins in fission and fusion. Initially, researchers found that overexpression of MID49 or MID51 caused mitochondrial fusion, elongating mitochondrial tubules projecting out of a dissociated perinuclear network (Palmer et al., 2011). Afterward, they found that the fusogenic effect was due to sequestration and inhibition of DRP1, allowing unchecked mitochondrial fusion by MFN1 and MFN2 alongside peroxisome fusion in mouse embryonic fibroblasts. They proposed that MID49 and MID51 could function in mitochondrial fission under common conditions (Palmer et al., 2013). Recent patient research proved this hypothesis further. The MID49 defect elevated mitochondrial fusion frequency and ragged-red fibers in patient’s muscles (Bartsakoulia et al., 2018). Surprisingly, the mtDNA levels of patient fibroblasts increased, along with the mildly increased OXPHOS. The author proposed that this is due to the intact compensate mechanism of the mitochondria, which allowed mitochondrial fusion to happen, thus fulfilling the energic need of cells (Bartsakoulia et al., 2018). Besides, an E3 ubiquitin ligase membrane-associated ring-CH finger protein 5 (MARCH5) could degrade MiD49, promoting mitochondrial fusion as a stress-response mechanism (Xu et al., 2016). MiD49 and MiD51 regulate cytochrome C release and then activate the Bax/Bak pathway. Loss of MiD49 and MiD51 prevents cristae from remodeling during apoptosis (Otera et al., 2016). Those pieces of evidence indicate that MiD49 and MiD51 are upstream players in mitochondrial fission, acting as the first-line responders upon apoptotic stress.

### Optic Atrophy 1

OPA1 is a mitochondrial dynamin-like GTPase that localizes to the mitochondrial inner membrane. *OPA1* comprises 32 exons and spans more than 40 kb. The deduced protein from *OPA1* contained 978 amino acids, with a size of about 120 kDa. OPA1 binds membranes enriched in negatively charged phospholipids, such as cardiolipin, and promotes membrane tubulation. OPA1 itself has a basal rate of GTP hydrolysis, which enhances its association with negatively charged phospholipids. OPA1 transfers into highly ordered oligomers when associated with lipids. OPA1 assembles on the lipid tubule surface, forming a protein-membrane structure similar to those of typical dynamins. Those shreds of evidence proved that OPA1 could stimulate higher-order membrane assembly, promote GTP hydrolysis, and transform membranes into tubules (Ban et al., 2010). OPA1 is also a pivotal player during apoptosis, and it is essential in remodeling cristae and releasing cytochrome C. Oligomerization of OPA1 regulates apoptosis by maintaining the tension of cristae connections (Frezza et al., 2006). Intrinsic apoptotic signals cause dissociation of OPA1 oligomers and release cytochrome C as the caspase activator into the intermembrane space. This process is also crucial in maintaining the mtDNA genome (Amati-Bonneau et al., 2008; Elachouri et al., 2011). OPA1 has ten isoforms. The isoform of OPA1 containing exon 4b could anchor the mitochondrial nucleoids to the inner membrane, whereas isoforms containing exon 4 are essential in upholding the membrane potential (Olichon et al., 2007; Elachouri et al., 2011). The long isoform of OPA1 (L-OPA1) is responsible for locking the intermembrane space inside cristae. After disassembly of L-OPA1–containing complexes, cytochrome c release leads to mitochondrial fragmentation (Jiang et al., 2014). OPA1 interacts with OMA1 zinc metallopeptidase (OMA1) and Mitochondrial-Escape 1-like 1 (YME1L1) to function correctly and maintain the L-OPA1 status.

Conversely, knockout of both Oma1 and Yme1l in mouse cardiomyocytes prevents the conversion of L-Opa1 to S-Opa1 forms and restores the standard mitochondrial architecture, in addition to protecting Yme1l mutant mice from cardiomyopathy and early death. Functional mitochondrial fusion mediated by L-OPA1 preserves cardiac function, and mitochondrial fragmentation may trigger dilated cardiomyopathy and heart failure (Wai et al., 2015). Fresh new research found that knockout of the phosphatidyl glycerophosphate synthase PSG1 causes cardiolipin reduction, thus rescuing mitochondrial fragmentation caused by OPA1 dysfunction (Cretin et al., 2021). OPA1 is another major player in maintaining the mitochondrial architecture, regulating apoptosis, and sustaining the homeostasis of mitochondrial fusion and fission.

### Mitofusin 1 and Mitofusin 2

MFN1 (Mitofusin 1) and MFN2 (Mitofusin 2) were initially discovered as the human homolog of the Drosophila and yeast protein fuzzy onion (Fzo), which regulate mitochondrial fusion. Fzo is crucial for spermatogenesis. Expression of Fzo is tightly restricted to the male germline, and it promotes the mRNA accumulation in both sperm cells and spermatids (Hermann et al., 1998; Hwa et al., 2002). The crystal structure of MFN1 showed that it has a GTPase domain and a C-terminal tail. A three-helix bundle that extends from the GTPase was determined, along with another extending from the C-terminal domain, together forming a classical configuration of the bacterial dynamin-like protein. MFN1 forms a dimer when GTP is attached and plays a role in the clustering of vesicles, including the membrane-anchoring GTP-binding domain, which requires undisrupted GTP hydrolysis. As a result, MFN1 tethers mitochondrial outer membranes *via* a nucleotide-dependent dimerization (Qi et al., 2016). Under anaerobic conditions, mitochondrial elongation is mainly regulated by SIRT1-mediated MFN1 deacetylation, while MFN2 induces mitochondrial fusion by facilitating ER–mitochondrial contact sites (Basso et al., 2018).

MFN2 was first designated as KIAA0214, containing 19 exons; the protein encoded by *MFN2* comprised 757 amino acids, containing an ATP/GTP-binding motif. Similarly, MFN2 has a GTPase domain and a C-terminal tail, sharing approximately 60% of the identity of MFN1. MFN2 targets mitochondria with a predicted bipartite transmembrane domain (Santel and Fuller, 2001). Knockdown of Mfn2 in rat myotubes reduced glucose oxidation by 30%. Inhibition of Mfn2 also leads to diminished mitochondrial membrane potential and aerobic cellular respiration (Bach et al., 2003). The authors further found that MFN2 was repressed under obesity. MFN2 expression was 39% lower in obese rats and 43% lower in obese humans than in lean controls (Bach et al., 2003). Obesity-induced MFN2 repression was also associated with the decline of mitochondrial ETC complexes I, II, III, and V (Pich et al., 2005). The promoters of apoptosis, BAX, and BAK play roles in mitochondrial fusion and regulate MFN2. BAX activates mitochondrial fusion by assembling MFN2, redirecting to the submitochondrial area, and alternating its GTP-binding state (Karbowski et al., 2006). Overexpression of *Mfn2* initiates apoptosis in rat vascular smooth muscle cells and prevents neointima formation after angioplasty. Attenuation of Mfn2 protects vascular muscle cells from damage by ROS (Guo et al., 2007). The authors further found that the proapoptotic effect MFN2 encaptured was mediated by the Akt signaling pathway (Guo et al., 2007). The location where MFN2 was enriched was then found to be the ER–mitochondrial interface. Interfering Mfn2 in Hela cells disrupted ER morphology and loosened mitochondrial–ER junctions, reducing calcium uptake upon stimuli (de Brito and Scorrano, 2008). Experiments in human T lymphocytes further proved this hypothesis. MFN2 increased buffering of intracellular Ca^2+^ (Luchsinger et al., 2016). MFN2 has to be precisely modified to achieve its normal function as well. Phosphorylated MFN2 was found as a parkin receptor in eliminating impaired mitochondria, indicating the quality controller role of MFN2 (Chen and Dorn, 2013). The molecular analysis determined that MFN2 achieves normal function *via* the rightful peptide–peptide interaction. The PINK1 kinase-mediated phosphorylation of MFN2 through ser378 is adjacent to the determinant of MFN2 activity interactions of met376 and his380 with Asp^725^ and Leu^727^ (Rocha et al., 2018). Mouse *Mfn2* mutant lacking the Pink1 phosphorylation sites inhibited mitochondrial Parkin translocation, repressing mitophagy (Gong et al., 2015). Furthermore, manipulating mitofusin conformations by an engineered cell-permeant mini peptide can reverse mitochondrial abnormalities in human fibroblasts and neurons (Franco et al., 2016). Overall, mitofusins are the central player in maintaining mitochondrial fusion and fission equilibrium, thus keeping mitochondria in a dynamic homeostasis to achieve normal cellular respiratory function.

### Solute Carrier Family 25 Member 46

SLC25A46 was first found to be one of the fourteen solute carrier proteins in the central nervous system (Haitina et al., 2006). The protein encoded by *SLC25A46* comprising 418 amino acids was the homolog of yeast Ugo1 in humans. Ugo1 is a mitochondrial solute in the outer membrane that acts as a fusion factor. SLC25A46 was integrated inside the outer mitochondrial membrane (Abrams et al., 2015). There is a hypothesis that SLC25A46 acts as a transporter across the outer membrane or as a protein adaptor resembling Ugo1 (Vásquez-Trincado et al., 2016). However, human SLC25A46 failed to rescue ugo1 deletion in *S. cerevisiae*. Mouse Slc25a46 protein has an approximately 100-residue N-terminal domain, followed by three tandem repeats of similar size. Each contains two transmembrane domains separated by a large loop, along with a distinct Px (D/E)xx (R/K) motif. The exact function of SLC25A46 is yet to be explored. SLC25A46 interacts with MFN2 and OPA1 and may combine with the cristae-restructuring protein MIC60 (Mitofilin) beside the ER–mitochondrial contact site. Besides, abrupted cristae have been determined in multiple disease models carrying the *Slc25a46* mutation (Abrams et al., 2015; Duchesne et al., 2017; Li et al., 2017). Janer and colleagues found SLC25A46 loss of function secondarily altered ER morphology, leading to premature cellular senescence. SLC25A46 also coordinated with the ER membrane protein complex EMC and altered phospholipid composition within mitochondria. The authors proposed that SLC25A46 plays a role in a mitochondrial–ER interface and facilitates lipid transfer; dysfunction of SLC25A46 altered mitochondrial dynamics, finally leading to cell death (Janer et al., 2016). The deletion of mouse and zebrafish *Slc25a46* leads to premature death, severe mitochondrial dysfunction, and hyperfused mitochondria (Abrams et al., 2015; Li et al., 2017). However, whether the hyperfusion of mitochondria in *Slc25a46* mutants is primary or secondary is still elucidated.

## Human Neurological Diseases Caused by Dynamic Dysfunction of Mitochondria

Changes in mitochondrial function and morphology are constantly studied in humans as disease triggers, and abnormal mitochondrial dynamics have been implicated as an early event in the pathogenesis of many diseases. Cardiac, metabolic, kidney, and neurological disorders can be linked to dynamic dysfunction of mitochondria. However, mitochondrial dysfunction of fission and fusion are mostly secondary under those conditions. Herein, we summarized disorders caused by mutation of mitochondrial dynamic–regulating genes primarily (Table 1). The most reported disorders caused by mitochondrial dynamic–controlled gene mutations are OPA1-related autosomal dominant atrophy and MFN2-related Charco–Marie–Tooth diseases (CMT). Nevertheless, DRP1, INF2, MIEF1, MIEF2, and SLC25A46 play essential roles, especially in neurological disorders.

**TABLE 1:** Primary disorders caused by mutation of mitochondrial dynamic–regulating genes.

Genes	Phenotype	Inheritance	References	Omim ID
DRP1	Encephalopathy, lethal, due to defective mitochondrial peroxisomal fission 1	AD, AR	Waterham et al. (2007); Yoon et al. (2016)	614,388
	Optic atrophy 5	AD	Gerber et al. (2017)	610,708
	Refractory epilepsy	AD	Vanstone et al. (2016)	N/A
	Mitochondrial cardiac encephalopathy	AD	Vandeleur et al. (2019)	N/A
INF2	Focal segmental glomerulosclerosis	AD	Brown et al. (2010)	613,237
	Charcot–Marie–Tooth disease, dominant intermediate E	AD	Boyer et al. (2011)	614,455
MIEF1	Singular late-onset optic neuropathy	AD	Charif et al. (2021)	N/A
MIEF2	Combined oxidative phosphorylation deficiency 49	AR	Bartsakoulia et al. (2018)	619,024
OPA1	Autosomal dominant optic atrophy	AD	Alexander et al. (2000); Delettre et al. (2000); Pesch et al. (2001)	165,500
	Optic atrophy and sensorineural deafness	AD	Amati-Bonneau et al. (2005); Li et al. (2005)	125,250
	Behr syndrome	AR	Schaaf et al. (2011); Bonneau et al. (2014)	210,000
	Mitochondrial DNA depletion syndrome 14	AR	Spiegel et al. (2016)	616,896
	Normal-tension glaucoma	AR	Aung et al. (2002)	606,657
MFN2	Charcot–Marie–Tooth neuropathy type 2A	AD, AR	Züchner et al. (2004); Kijima et al. (2005); Guillet et al. (2010); Polke et al. (2011)	609,260
	Hereditary motor and sensory neuropathy with optic atrophy	AD	Züchner et al. (2006)	601,152
SLC25A46	Hereditary motor and sensory neuropathy, type VIB	AR	Abrams et al. (2015)	616,505
	Progressive myoclonic ataxia with optic atrophy and neuropathy	AR	Charlesworth et al. (2016)	N/A
	Pontocerebellar hypoplasia, type 1E	AR	Wan et al. (2016); van Dijk et al. (2017); Braunisch et al. (2018)	619,303

### Charcot–Marie–Tooth Diseases 2A

CMT2A occurs due to heterozygous mutation of MFN2, featured as sensory and motor neuropathy of peripheral nerves (Züchner et al., 2004). Unlike CMT, CMT2A is an axonopathy with no or slight reduction of neuronal conduction velocity (Vásquez-Trincado et al., 2016). The typical clinical features include hammertoes, foot drop, distal limb muscle weakness and atrophy, hyporeflexia, or areflexia. MFN1 and MFN2 interact with Miro and Milton proteins that form the molecular complex linking MFN2 to kinesin motors. Mutation in MFN2 affects this interaction, leading to impairment in axonal mitochondrial transport (Adebayo et al., 2021). Early onset is often associated with more severe cases, resembling dominant optic atrophy caused by OPA1 mutations (Hamedani et al., 2021). Besides, deletion of *MFN2* is perinatally lethal to the embryo in both murine and canine models, likely due to lack of protein stability.

### Dominant Optic Atrophy

OPA1-related DOAs commonly diagnosed in early childhood are characterized by progressive bilateral visual loss, color vision loss, visual field defects, optic nerve atrophy, and optic disc excavation (Kjer et al., 1996). DOA plus is associated with central or peripheral neuronal defects featured by deafness, ataxia, myopathy, and progressive external ophthalmoplegia (Amati-Bonneau et al., 2005; Amati-Bonneau et al., 2008; Yu-Wai-Man et al., 2010). Pathological studies suggested retinal ganglion cell degeneration and myelin loss of the optic nerve (Alexander et al., 2000; Kim et al., 2005). There have been reports of variable phenotypes and incomplete penetrance. DOA is the most reported inherited optic atrophy with a 1/12,000–1/50,000 prevalence (Kjer et al., 1996). Most deleterious OPA1 mutations are likely due to haploinsufficiency, resulting in dominant-negative effects (Stuppia et al., 2015). DOA plus can show mitochondrial deletion in some muscle fibers with OXPHOS dysfunction following OPA1 loss of function (Chen et al., 2010). Consistent with their protein function, unsurprisingly, DOA plus and CMT2A associated with MFN2 share many features; they both affected motor and sensory neurons. Due to the relatively long axons, these neurons are considered sensitive to mitochondrial dynamics dysfunction.

### Encephalopathy Due to Mitochondrial and Peroxisomal Fission 1

Patients affected by DRP1 mutations were initially reported to be suffering from lethal encephalopathy due to mitochondrial and peroxisomal fission 1. Symptoms of this desperate disorder include microcephaly, abnormal brain development, refractory epilepsy, optic atrophy, persistent lactic acidemia, and elevated plasma very long-chain fatty acids (VLCFA) (Fahrner et al., 2016; Sheffer et al., 2016). Abnormal gyral patterns, bilateral cerebral volume loss, demyelination, thinning of the corpus callosum, and T2-weighted hyperintense lesions in the cortex were visible on MRI in a certain proportion of the patients (Waterham et al., 2007). The number of peroxisomes of the patient’s cells was reduced while the sizes varied significantly. The severity of this disorder varies, but most of the patients die in early childhood. Fluorescence microscopy revealed elongated mitochondria concentrated around the nucleus (Waterham et al., 2007). Mitochondrial respiratory chain enzyme activity showed decreased complex IV activity and reduced ATP production (Sheffer et al., 2016). Cardiac involvement is also frequently observed in patients with DRP1 mutations. Dilated left ventricle, reduced ejection fraction, and reduced shortening fraction were visible on the echocardiogram. Post–Morten electronic microscopy found filamented mitochondria inside the patient cardiomyocytes.

### Alzheimer’s Disease

Amyloid protein β accumulates in the brains of patients with AD (Huang and Mucke, 2012). Disruption of dynamic mitochondrial homeostasis could be a crucial factor of neuronal apoptosis, thus leading to abnormal neurodevelopment. Nitric oxide (NO) was thought to be involved in the process of Amyloid protein β production. It is an essential regulatory factor that regulates mitochondrial division through S-nitrosylated drp1 (SNO-DRP1), reducing synapses and damaging neurons. It regulates mitochondrial division, which reduces synapses and damages neurons. However, cysteine mutation can prevent DRP1 nitrosylation and eliminate its neurotoxicity. Furthermore, nitrosylation can eliminate its neurotoxicity. In addition, SNODRP1 is also highly expressed in the brains of patients with AD (Cho et al., 2009). Researchers found that primary neurons of the Tau^−/−^ mouse transfected with truncated Tau showed fragmented mitochondria. They further found that a significant reduction of OPA1 accompanied mitochondrial fragmentation. They concluded that the Tau could impair mitochondrial dynamics by reducing OPA1 levels, leading to mitochondrial impairment in AD (Pérez et al., 2018).

### Parkinson’s Disease

Disruption of mitochondrial activity may be associated with PD, especially under the dysfunction of PINK1 and Parkin. PINK1 and Parkin are localized in the mitochondria (Wang et al., 2021). PINK1 protects against mitochondrial dysfunction under stress by phosphorylating mitochondrial proteins. PINK1 is also involved in eliminating damaged mitochondria *via* mitophagy by mediating activation and translocation of Parkin; it targets Parkin to dysfunctional mitochondria with low membrane potential through the phosphorylation of MFN2 (Chen and Dorn, 2013). As an E3 ubiquitin ligase, Parkin could induce the ubiquitination of MFN1, MFN2, FIS1, and DRP1; fragmented mitochondria was initiated once the functions of PINK1 and Parkin are damaged (Ziviani et al., 2010; Wang et al., 2011). Thus, PD pathology is somehow related to mitochondrial dynamics; however, it tends to be secondary rather than the primary cause.

### Huntington’s Disease

Over-fragmented mitochondria were identified in patients with HD and animal models, leading to a decreased OXPHOS rate (Bossy-Wetzel et al., 2008). In HD, mitochondrial abnormalities, morphological changes, and dysfunction are visible. Hungtitin protein (HTT), located in mitochondria, might be the cause of mitochondrial fragmentation. HTT mutants could accumulate in the body and trigger DRP1 dysfunction, leading to mitochondrial transport abnormalities and ultimately leading to neuronal apoptosis (Song et al., 2011; Shirendeb et al., 2012). Of note, long CAG repeats in HTT could promote the age-dependent expansion of pathogenic mtDNA heteroplasmy in HD lymphoblasts (Wang et al., 2021). Thus, they concluded that mtDNA quality is declining along with the HD’s process, indicating a role of HTT in mtDNA quality control. Controversially, the mitochondrial membrane was found to impact the HTT aggregation as well (Adegbuyiro et al., 2021). Collectively, the mitochondrial dynamic is heavily involved in HD; however, it is mainly subordinate.

## Emerging Therapeutic Approaches Targeting Primary Mitochondrial Fission–Fusion Abnormalities

Previous studies that aimed to intervene in mitochondrial fusion and fission worked either by overexpression or silencing the dysfunctional genes. Promising results from laboratories came out with pharmaceutical potential. DRP1 has to translocate to mitochondria to ensure fragmentation, which requires rightful posttranslational phosphorylation. PKA, Cam kinase, and Pim1 mediate phosphorylation in Drp1 Ser^637^. Phosphorylated rat Pim 1 increases Drp1Ser^637^ phosphorylation and inhibits Drp1 localization to the mitochondria, protecting rat cardiomyocytes from P53 upregulated modulator apoptosis (PUMA) (Din et al., 2013). A couple of years ago, Franco and colleagues reported that a mini-peptide derived from MFN2, capable of competing with endogenous peptide–peptide interactions hampering MFN1 and MFN2 into inactive conformations, transformed the latter into a more active construction, thus promoting mitochondrial fusion (Franco et al., 2016). However, this kind of peptide might still be costly to manufacture, hampering the clinical therapeutic use. Rocha and coworkers used *in silico* screening to identify a mitofusin agonist. They returned axonal mitochondrial traffic to normal in the sciatic nerves of MFN2 mutant mice and mitigated the dysmotility and fragmentation depolarization and clattering of mitochondria (Rocha et al., 2018). Most recently, Franco and collaborators reported a small molecule named MiM111, which can activate mutant-inhibited MFN2 and normalize neuromuscular function in CMT2A, further reversing axon and myocyte atrophy. They claimed that MiM111 was the first preclinical candidate treatment for CMT2A (Franco et al., 2020). Furthermore, as a widely used tool for gene therapy, adeno-associated virus (AAV)-mediated gene replacement or editing is now undergoing clinical translation. Using AAV-*Slc25a46*, Yang and colleagues ameliorated SLC25A46-related mitochondrial hyperfusion in a murine model and recovered the movement disorder and sciatic nerve demyelination, extending the longevity of the *Slc25a46* ^−/−^ mouse (Yang et al., 2020).

On the other hand, inhibitors of mitochondrial fission may hold promise as therapeutic targets to treat patients with mitochondrial over-fission, providing a protective effect for mitochondria viability under certain circumstances, thus attracting researcher’s interest. Mdivi (Cassidy-Stone et al., 2008), P110 (Qi et al., 2013), and Dynasore (Macia et al., 2006) are mitochondrial fission inhibitors. Cassidy and colleagues found that Mdivi inhibited the assembly of Drp1 and its GTPase enzymatic activity *in vitro* by binding the outside of the GTPase domain, thus inhibiting GTPase activity. However, Mdivi treatment does not impact Drp1 expression (Xie et al., 2013). Moreover, multiple studies investigated the inhibitor function of Mdivi *in vitro* and proved its protecting role of mitochondrial function under apoptotic stress (Tang et al., 2013; Wappler et al., 2013). Furthermore, Mdivi could rescue both mitochondrial over-fission and improve mitochondrial function in the CRND8 AD mouse model (Wang et al., 2017) and multiple mouse models of cerebral ischemia (Flippo et al., 2018) or organ injury (Tábara et al., 2014; Rogers et al., 2021). Qi and colleagues developed a Drp1 inhibitor named P110 and found that P110 decreases Fis1 expression and reduces excessive mitochondrial fission in cultured neurons. Furthermore, P110 reduced ROS production, improving mitochondrial membrane potential and mitochondrial integrity (Qi et al., 2013). Marcia and coworkers identified Dynasore from 16,000 small molecules as a fission inhibitor. They found that Dynasore interferes with the GTPase activity of Dynamin1, Dynamin 2, and Drp1 (Macia et al., 2006). Gao et al. further investigated the protective effects of Dynasore against ischemia/reperfusion injury in mice. Dynasore increased cardiomyocyte survival and reduced the depletion of cellular ATP (Gao et al., 2013).

To date, there is no clinical trial registered or any human case reported yet using Mdivi-1. The main reason is that the mitochondrial over-fission mouse model is often lethal to the embryo (Chen et al., 2003; Davies et al., 2007). Therefore, researchers could not test the therapeutic and side effects on animal models, not to mention trials on humans. Later on, mouse models capable of surviving after birth carrying the MFN2 point mutation were built (Detmer et al., 2008; Cartoni et al., 2010). However, until recently, no report had tested the therapeutic effect of the mitochondrial fission inhibitors on these models. More therapeutic explorations are needed to determine their protecting efficacy from mutant protein-induced neuronal damage and promising candidates to treat patients.

## Mitochondrial Dynamics and Reproduction

Female reproductive mitochondria are associated with oocytes and embryos because they donate ATP *via* OXPHOS (May-Panloup et al., 2016). The physiological processes include spindle assembly, chromosome separation, oocyte maturation, fertilization, and embryonic development (Rodríguez-Varela and Labarta, 2020). However, mitochondrial function in female germ cells appears to be more than superficial.

Mitochondrial replication is constantly in progress during oogenesis, and the number of mitochondria increases as oocytes mature (Jansen and de Boer, 1998). The mtDNA copy number maintains a relatively stable state in the mature oocyte and early embryo in many mammals, including humans (Pikó and Taylor, 1987; Ebert et al., 1988; Kameyama et al., 2007; Hashimoto et al., 2017). Therefore, mitochondrial replication will be suspended during this time, resulting in relatively steady levels of mitochondria (Jansen and de Boer, 1998; Collado-Fernandez et al., 2012). Moreover, before the blastocyst stage, the mature oocyte and embryonic metabolism primarily depend on pyruvate by OXPHOS (Collado-Fernandez et al., 2012; Bradley and Swann, 2019). Consequently, the number and quality of mitochondria in oocytes and embryos must be sufficient to provide enough energy for embryonic development. These mitochondria must be relatively evenly distributed to blastomeres until mitochondrial biogenesis resumes in blastomeres (May-Panloup et al., 2021). Thus, mitochondria quantitative and morphological abnormalities in the oocyte and early embryo, or the defective distribution of mitochondria in blastomeres, will lead to reduced OXPHOS, resulting in fertilization failure and embryonic development dysfunction (Van Blerkom et al., 1995; Van Blerkom et al., 2000).

In contrast to somatic cells, oocytes and early embryos have radically different mitochondrial morphology and submitochondrial structure. The cytoplasm comprises many spherical mitochondria with parallel or vaulted cristae and pale matrices in the early stages of development. These mitochondria gather with other organelles around the nucleus to form the Balbiani’s vitelline body. In mature oocytes, mitochondria are minor and present with a round or oval shape with arched cristae. Most mitochondria form unique structures with the tubular membrane of the smooth endoplasmic reticulum (M-SER aggregates) and vesicles (MV complexes) (Szöllösi et al., 1986; Motta et al., 1988). These structures are supposed to reserve material and membranes for subsequent fertilization and embryo development (Motta et al., 2000). Mitochondrial morphology does not change significantly in the zygote and 2-cell embryo after fertilization (round or oval mitochondria that are 0.4–0.6 μm in length). Longer mitochondria (1.5–2.5 μm) and more abundant cristae are observed by transmission electron microscopy in 4-cell embryos, indicating increased mitochondrial activity (Motta et al., 2000). The mitochondria in 6- to 8-cell embryos become much more prolonged (2.5 μm), and mitochondrial cristae are more abundant (Sundström et al., 1981; Motta et al., 2000). Recovery of mtDNA replication first occurs in trophoblastic cells of the blastocyst, which coincides with a significant increase in embryo energy demand (Houghton, 2006; St. John et al., 2010; St. John, 2014).

From here, we see that mitochondria maintain a spherical or elliptic shape in the mature oocyte and the early embryo, with a maximum length of 2.5 um, and sparse, immature cristae. The typical elongated or rod-shaped mitochondria with abundant transverse cristae were not observed in the oocyte or embryo. Consequently, mitochondrial activity is low, and oxygen consumption and ATP production are also reduced due to the undifferentiated mitochondrial morphology (Van Blerkom et al., 1995). Therefore, the mitochondria’s total number and function, especially the mitochondria with high membrane potential (usually reflecting high mitochondrial activity), are essential for embryonic development (Au et al., 2005). In addition, the spatial location of mitochondria also seems to be related to the embryo’s developmental competence. Mitochondria must be distributed to cytoplasmic locations with high energy requirements to support critical events in oocyte maturation and embryonic development, such as pronounced mitochondrial aggregation (Van Blerkom et al., 2000). Mitochondria maintaining the low active forms (round mitochondria with sparse cristae) in oocytes and embryos most of the time is beneficial to reducing ROS production as much as possible (Ramalho-Santos et al., 2009) and thus minimizes oxidative damage to the oocytes and embryos. The mitochondria change the morphology in response to the increased demand for ATP only when a particular event occurs (cell division) (Van Blerkom, 2011). Therefore, either mitochondrial dysfunction or excessive function in oocytes and embryos will result in embryonic development impairment.

The role of mitochondrial dynamic–related proteins in germ cells and embryos has been studied in some knockout mice and specific knockout mouse germ cells and embryos. The specific deletion of *Drp1* in oocytes leads to aggregation of malformed fusion mitochondria, impaired calcium oscillation, secretory function, meiosis of oocytes, and female mice infertility caused by oocyte maturation and ovulation disorders in an age-dependent way (Udagawa et al., 2014). This infertility could correlate with human embryo fragments on the third day (Otasevic et al., 2016). In addition, the use of Mdivi-1, a DRP1 inhibitor, reduced the formation of pig blastocysts with decreased mitochondrial membrane potential and increased ROS (Yeon et al., 2015). *Mfn1/Mfn2* double knockout oocytes showed mitochondrial structural damage, including reduced mitochondrial cristae and decreased matrix density, resulting in the arrest of oocyte development and impaired oocyte–granulosa cell interaction (Zhang et al., 2019a). *Mfn2* knockout mouse oocytes displayed decreased oocyte maturation and fertilization rates and an alteration in mitochondrial distribution and spindle morphology (Liu et al., 2016), which further suggested the role of mitochondrial dynamics in regulating chromosome separation in oocytes. In addition, siRNA-induced reduction of *Mfn2* expression diminished the rate of blastocyst formation in mice and the number of embryos passing through the third cell division (Zhao et al., 2015). Mitochondrial fusion may be required to support this milestone event as activating the embryonic genome. *Mfn2* deletion in oocytes causes female infertility resulting from mitochondrial dysfunction, oocyte maturation, and follicular development blocking (Zhang et al., 2019b). Embryos that survived *Mfn2* knockout showed decreased levels of ATP, mitochondrial membrane potential, and mtDNA deficiency and increased levels of mitochondrial apoptosis (Zhao et al., 2015), further suggesting that mitochondrial fusion is essential for maintaining oocyte and embryonic development. In contrast, the irregular mitochondrial distributions, including mitochondrial aggregation and ER coaggregation and increased contact between mitochondria and mitochondria and mitochondria and the ER, were detectable in overexpression of *Mfn1* or *Mfn2* oocytes. The unusual behavior of mitochondria and the ER results in impaired calcium homeostasis and abnormal chromosomal segregation during meiosis (Wakai et al., 2014). Consequently, similarly, deficiency or overexpression of mitochondrial dynamic–related proteins will develop from mitochondrial morphological malformations, unusually low or high mitochondrial activity, increased ROS production, and disturbance of intracellular signal molecules.

Therefore, mitochondrial fission and fusion homeostasis are of great consequence to the maintenance of oocyte development, maturation, and embryo development. How can we improve the oocyte and embryo development ability by modulating mitochondrial dynamics, especially improving the reproduction ability of patients with oocyte maturation disorders or embryo development arrest? When *in vitro* culture conditions are altered, mitochondrial morphology, distribution, and function may be affected (Barnett et al., 1997; Squirrell et al., 2001). The addition of melatonin in the medium of *in vitro* maturation has been shown to promote the maturation of immature oocytes and subsequent embryonic development by increasing the production of ATP, reducing intracellular ROS generation, and lowering calcium levels (Zou et al., 2020).

According to research, adding proper levels of melatonin to the culture medium resulted in the best oocyte maturation, fertilization, and blastocyst formation rates. Too low or too high of a melatonin concentration could not achieve the best effects (Li et al., 2019). Mitochondrial fission and fusion homeostasis is the critical factor in ensuring the moderate mitochondrial activity in oocytes and embryos that can maintain the normal development of embryos and avoid oxidative damage. As more molecules or drugs are discovered and confirmed, molecules regulating mitochondrial dynamics may be a future target for improving female fertility.

## Conclusion

**FIGURE 1:**
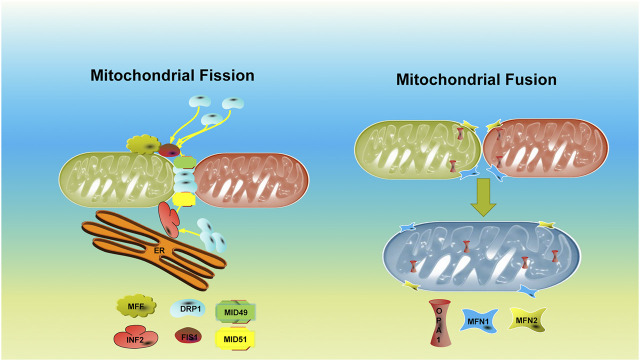
Schemes and fundamental regulators of mitochondrial dynamics.

In summary, the homeostasis of mitochondrial dynamics is a multiprotein regulation physiological process (Figure 1). Therefore, abnormalities of relevant genes or proteins in somatic cells or germ cells will lead to disorders of the mitochondrial dynamic balance due to impaired mitochondrial morphology and function, leading to organ dysfunction and diseases. DRP1, MFN1, and MFN2 are closely related to female reproduction. Targeted treatment of the gene or protein may be a therapeutic method for mitochondrial dynamic–related diseases. However, there is still further to go from experimental research to clinical application.
